# Managing healthcare delivery system to fight the COVID-19 epidemic: experience in Japan

**DOI:** 10.1186/s41256-020-00149-0

**Published:** 2020-05-13

**Authors:** Ruoyan Gai, Makoto Tobe

**Affiliations:** 1 National Institute of Population and Social Security Research, Tokyo, Japan; 2 Japan International Cooperation Agency, Tokyo, Japan

## Abstract

Amid the global pandemic of a novel Coronavirus Disease 2019 (COVID-19), healthcare delivery system is being stretched. In Japan, rapid spread of the epidemic brings hospitals to the brink of exhaustion. This commentary aims to briefly review related policies of Japan in managing healthcare delivery system. Among the relevant actions, strengthening the hospitalized care is emphasized to save lives. Despite of limitations, the policies show a success in preventing a collapse of healthcare delivery system and skyrocketing mortality from happening so far. On the other hand, huge concerns remain if the infections continue to rapidly increase. The experience in Japan indicates the urgency of planning of healthcare delivery system, mobilizing all relevant social sectors by consensus, and guiding people with calm manner based on the best shared knowledge and evidences.

## Background

**Fig. 1:**
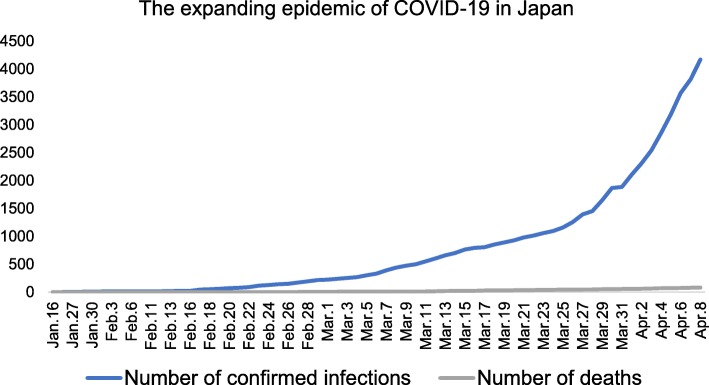
the expanding epidemic of COVID-19 in Japan. Data derive from the daily report of Ministry of Health, Labour and Welfare of Japan

The pandemic of a novel Coronavirus Disease 2019 (COVID-19) caused by the severe acute respiratory syndrome coronavirus 2 (SARS-CoV2) has posed a severe global crisis. In Japan, the incidence is escalating, causing a large number of community transmission. As of April 8, 2020, the time point that the statement of emergency is initiated, the number of domestic confirmed infection cases reaches up to 4168, with 81 deaths (Fig. 1). The policies in Japan highlight the planning of healthcare delivery system, in particular the prevention of an “overshoot” in hospitals. To this end, a crucial lesson has been learned from Wuhan, China, where hospitals once melted down at the beginning as the result that patients were overwhelmingly seeking healthcare, and consequently those severe cases urgently needing inpatient care cannot be hospitalized. The exhaustion of healthcare delivery system is one of the reasons for the incredibly high mortality rate in that period [1]. The overcrowded and contaminated environment of hospitals exposed a large population to high risk of the infection, further exacerbating the epidemic. Similar conditions now also occur in seriously hit countries such as Italy and Spain, where the crude mortality rate due to COVID-19 largely exceeds the global average level of 3.4% [2]. Confronting the epidemic, it should be noted that the capacity of healthcare delivery system is not unlimited and consequently it entails a series of policies to avoid a surge of the infected. This commentary focuses on adaptation of healthcare delivery system for the containment of COVID-19 in Japan, for knowledge sharing with the global society. The major related measures adopted in Japan are outlined.

## Guiding healthcare seeking

As COVID-19 is listed to be a designated infectious disease in the Infectious Diseases Control Law, the patients are rendered to the hospitalized care in the designated facility in general. On the other hand, the overwhelming healthcare seeking may lead to a collapse of healthcare delivery system as mentioned above. In this regard, the basic policies for COVID-19 control emphasized the hospitalized care for the severe / critical case based on previous experiences [3], and recommended people with mild flu-like symptoms to stay at home first and seek medical care after consulting the call center or a family doctor [4]. After the declaration of state of emergency, Tokyo metropolitan government decides to move patients with mild or no symptom (except the elderly, those with pre-existing health conditions and pregnant women) from hospitals to the assigned facilities to increase bed capacities with serious cases for COVID-19. To mitigate mortality and morbidity, it is worthy to note that the elderly, those with pre-existing diseases and pregnant women are specially considered for healthcare delivery, including detection and inpatient-based treatment, because of their vulnerability [3, 5].

## Planning the implementation of the detection test

In Japan, because of a limited laboratory capacity, priorities are given to determining cases requiring an inpatient care and tracing the cluster transmission, rather than to exploring the infection in the overall population through massive screening. Meanwhile, the test method based on polymerase chain reaction (PCR) has a limited accuracy [6], especially when the test is used for a large cohort of population. A relevant proportion of false positive is prone to cause spread of infection from these tested negative but truly positive, whereas a large number of false negative leads to unnecessary occupation of limited healthcare resources that could be otherwise for severe cases.

With regard to this, the implementation of detection test based on PCR has been scheduled by the call center for consultation, which was established within each health center of prefectures and government-designated cities right in early February 2020. The criteria of seeking consultation are stipulated and updated, with consideration of high-risk population, such as the elderly, those with pre-existing health conditions and pregnant women. The call-center-based consultation system plays a key role of guidance of healthcare seeking and referral. It also provides a clue to capture the overall epidemic scale in the country. As the laboratory capacity has gradually improved, the PCR test has been included into benefit package of the social health insurance schemes and offered under physician’s discretion since March 2020.

## Improving the capacity of healthcare delivery system

The capacity of healthcare delivery system is challenged by rapidly growing severe / critical cares [7]. In Japan, it is largely calling for wide negotiations and collaborations among the national and local governments, manufacturers and healthcare providers. Since emerging of COVID-19, the national government has urged local authorities to prepare to secure hospital beds, intensive care unit and medical equipment in hospitals, especially for those not originally designated and specialized for infectious diseases. Guidelines for infection control measures developed by the Ministry of Health, Labour and Welfare help to inform hospitals and health professionals. The government has also mobilized the manufacturing to ensure the supply chain of medical equipment and materials. On the other hand, it is reported that difficulties nevertheless remain in local authorities to offer the number of beds equivalent to the estimates of the expert panel assigned by the health ministry during the peak period of the epidemic [8]. Besides limited hospital beds and materials, a shortage of doctors, especially infectious disease specialists remain a huge concern [7, 9]. The high risk of iatrogenic infection and lack of support to child care during school closure may further deteriorate the situation.

## Suppressing the scale of the epidemic

The limitation in the capacity of healthcare delivery system suggest an urgency to suppress the scale of the epidemic. In Japan, routine hygiene behaviors and health literacy of self-motivated and well-informed public compose the first line of defeating COVID-19. Effective measures such as mask wearing [10], hand washing and health management are well penetrated in daily life. Since the outbreak, clusters and contacts are closely traced by the public health officers to generate the firsthand data source for evaluation by the expert panel of MHLW. Then a number of measures have been implemented with engagement of various stakeholders, including different governmental sectors, expert panels, local authorities, schools, industries and civil society, such as a nationwide school closure, promotion of telecommuting, staggered commuting time and voluntary restraints. Knowledge on pathological, epidemiological and clinical characteristics of the virus and disease and public health measures are constantly updated to the public. Social distancing is being reinforced. On the other hand, a lockdown as the most radical measure of social distancing may lag behind because of lack of legal force, suggesting the containment in Japan is largely relying on consensus, self-motivation and empowerment of the public.

## Boosting public understanding and consolidated trusts

As mentioned, underpinning the promotion of these policies are public understanding and consolidated trusts. As public health measures are not compulsory but voluntary in the country, the government is striving to get consensus and to consolidate the trusts from relevant stakeholders including local authorities and the public by providing information that is accurate, prompt and easy to understand and practice. The current policies also emphasize a thoughtful consideration of the growing concerns and fears in public. Especially at this moment when public anxiety and fear are spreading together with the growing number of infections, effective communicating channels to the public are necessary to promote public understanding and encourage calmness.

## Conclusion

In Japan, preventing the exhaustion of healthcare delivery system and softening the spike of the epidemic are regarded to be of paramount importance. These policies have limitations in testing, resource mobilizing and social distancing. However, the number of death cases since the emerging of COVID-19 suggests these priority-based measures have partly contributed to deferring the occurrence of the spike, adapting healthcare delivery system and saving lives so far. It should be recognized that, on the other hand, concerns remain in the capacity of healthcare delivery system if the epidemic continues to rapidly progress. To this end, dealing with the crisis further requires an agile and coordinative leadership to consolidate consensus and trusts. The experiences in Japan to date indicates the importance of (1) preparing healthcare delivery system for the epidemic, (2) mobilizing all relevant stakeholders and social sectors by the consensus, and (3) guiding people with calm manner based on the best shared knowledge and evidence, and all that are attributable to contingency planning and preparedness at early stage.

## Data Availability

Not applicable.

## Abbreviations

COVID-19: Coronavirus Disease 2019
MHLW: Ministry of Health, Labour and Welfare
PCR: Polymerase chain reaction
SARS: The severe acute respiratory syndrome
SARS-CoV2: The severe acute respiratory syndrome coronavirus 2
WHO: World Health Organization
